# Cooperative Genome-Wide Analysis Shows Increased Homozygosity in Early Onset Parkinson's Disease

**DOI:** 10.1371/journal.pone.0028787

**Published:** 2012-03-12

**Authors:** Javier Simón-Sánchez, Laura L. Kilarski, Michael A. Nalls, Maria Martinez, Claudia Schulte, Peter Holmans, International Parkinson's Disease Genomics Consortium, Wellcome Trust Case Control Consortium, Thomas Gasser, John Hardy, Andrew B. Singleton, Nicholas W. Wood, Alexis Brice, Peter Heutink, Nigel Williams, Huw R. Morris

**Affiliations:** 1 Section of Medical Genomics, Department of Clinical Genetics, VU University Medical Centre, Amsterdam, The Netherlands; 2 MRC Centre for Neuropsychiatric Genetics and Genomics, Cardiff University School of Medicine, Cardiff, United Kingdom; 3 Laboratory of Neurogenetics, National Institute on Aging, National Institutes of Health, Bethesda, Maryland, United States of America; 4 Inserm, UMR 1043, Toulouse, France; 5 Paul Sabatier University, Toulouse, France; 6 Department of Psychological Medicine & Neurology, Cardiff University School of Medicine, Cardiff, United Kingdom; 7 Department for Neurodegenerative Diseases, Hertie Institute for Clinical Brain Research, and DZNE, German Center for Neurodegenerative Diseases, Tübingen, Germany; 8 Department of Molecular Neuroscience, UCL Institute of Neurology, London, United Kingdom; 9 Université Pierre et Marie Curie-Paris6, Centre de Recherche de l'Institut du Cerveau et de la Moelle épinière, UMR-S975, Paris, France; 10 Inserm, U975, Paris, France; 11 Cnrs, UMR 7225, Paris, France; University of Florida, United States of America

## Abstract

Parkinson's disease (PD) occurs in both familial and sporadic forms, and both monogenic and complex genetic factors have been identified. Early onset PD (EOPD) is particularly associated with autosomal recessive (AR) mutations, and three genes, *PARK2*, *PARK7* and *PINK1*, have been found to carry mutations leading to AR disease. Since mutations in these genes account for less than 10% of EOPD patients, we hypothesized that further recessive genetic factors are involved in this disorder, which may appear in extended runs of homozygosity.

We carried out genome wide SNP genotyping to look for extended runs of homozygosity (ROHs) in 1,445 EOPD cases and 6,987 controls. Logistic regression analyses showed an increased level of genomic homozygosity in EOPD cases compared to controls. These differences are larger for ROH of 9 Mb and above, where there is a more than three-fold increase in the proportion of cases carrying a ROH. These differences are not explained by occult recessive mutations at existing loci. Controlling for genome wide homozygosity in logistic regression analyses increased the differences between cases and controls, indicating that in EOPD cases ROHs do not simply relate to genome wide measures of inbreeding. Homozygosity at a locus on chromosome19p13.3 was identified as being more common in EOPD cases as compared to controls. Sequencing analysis of genes and predicted transcripts within this locus failed to identify a novel mutation causing EOPD in our cohort.

There is an increased rate of genome wide homozygosity in EOPD, as measured by an increase in ROHs. These ROHs are a signature of inbreeding and do not necessarily harbour disease-causing genetic variants. Although there might be other regions of interest apart from chromosome 19p13.3, we lack the power to detect them with this analysis.

## Introduction

Parkinson's disease (PD) is an age-related neurodegenerative condition which causes a progressive L-DOPA responsive hypokinetic movement disorder related to nigro-striatal dopaminergic cell loss [1]. There is heterogeneity in the extent of non-motor symptoms and the presence of dystonia and L-DOPA related treatment complications. Autosomal dominant, recessive, common and rare variant genetic factors have been identified as being relevant to the development of PD [2]–[7]. The identification of these factors has informed clinical diagnosis, the study of disease heterogeneity, neuropathology and the understanding of the underlying pathogenic mechanisms. Furthermore, knowledge of genetic factors contributing to PD might enable the development of predictive testing and personalised treatments in the future. Age is the most certain risk factor for PD with the majority of patients developing disease after the age of 65 [8]. However, 3.6% of patients develop early-onset PD (EOPD) before the age of 45 and 1% develop their disease before the age of 40 [9]. Presumably, these outlying cases relate to the effects of exceptional genetic and/or environmental risk factors. Segregation analysis in PD indicates that there is an up to eight-fold increased risk of developing PD in siblings of patients with EOPD, supporting the effect of autosomal recessive genes [10], [11].

*PARK2* (parkin), *PARK7* (DJ-1) and *PINK1* (*PARK6*) have been identified as loci/genes that contain mutations causing an autosomal recessive form of the disease, based on mutation discovery in consanguineous families following homozygosity mapping and positional cloning [12]–[14]. Recently mutations in *ATP13A2*, *PLA2G6*, *FBXO7* and *SPG11* which cause a similar syndrome, pallido-pyramidal early onset parkinsonism, have also been identified using homozygosity mapping [15]. Pathogenic mutations in EOPD genes are not confined to familial or consanguineous patients. Screening of outbred EOPD patients has identified compound heterozygous and further homozygous mutations [16], [17]. Overall, 5% of EOPD cases have mutations in known autosomal recessive genes, with approximately half being homozygous and half being compound heterozygous [18].

Genome wide single nucleotide polymorphism (SNP) chips have been used to identify common risk alleles for typical sporadic PD [3]–[7]. However, they also provide the opportunity to identify homozygous runs in the genome [19], [20], shown to be abundant in ostensibly outbred populations [21]. This suggests that large-scale homozygosity mapping might be used to identify new genes in apparently outbred individuals with autosomal recessive disease, and to estimate the burden of recessive loci in a particular disease population [22]. We hypothesise that there are further autosomal recessive risk factors for EOPD and have performed genome wide homozygosity analysis, to determine the presence and extent of excess homozygosity in patients with early onset disease.

## Methods

### Participants and genotyping

DNA samples in this study were analysed as part of genome-wide association studies (GWAS) included in the International PD Genomics Consortium (IPDGC) meta-analysis published in the Lancet in February 2011 [23]. The authors are members of the consortium and consortium members are co-authors of this paper. The study represents a re-analysis of a part of the GWAS meta-analysis data (relating to early onset PD) and additional Cardiff EOPD samples were genotyped and included in this study, generated and analysed by our centre. Approval for this was given by the UK Research Ethics Committee Approval (REC for Wales 09/MRE09/35). Part of the data was generated by the Wellcome Trust Case Control Consortium 2 (WTCCC2). The authors have the permission and approval of both IPDCG and WTCCC2 to carry out this work and both IPDGC and WTCCC2 have approved this manuscript for submission for publication.

DNA samples from PD patients meeting Queen Square Brain Bank criteria with an age at onset (AAO) at or below 50 years (n = 1557 – France 466, Netherlands 286, Germany 239, USA 280, UK 286) (table 1) were collected and genotyped with Illumina HumanHap 550, Human660W-Quad, or Human1M-Duo beadchips (www.illumina.com), and had undergone some prior quality control procedures (QC). Following two rounds of QC aiming to unify the datasets (see supporting information S1 for details), consensus genotypic information for 412,212 unique SNPs was available for 1,445 EOPD cases and 6,987 controls (1958 British Birth Cohort (n = 1225, http://www.b58cgene.sgul.ac.uk), the British Blood Donor Service (n = 2510), US-American NINDS spousal and population controls (n = 750), the Rotterdam Study (n = 1559), and German controls from the KORA study and POPGEN project (n = 943)) (table S1). Detailed sample information is available elsewhere [3], [5]–[7].

**Table 1:** Samples and SNPs.

	Before QC			After QC (1)			After QC (2)		
Country	Cases	Controls	SNPs	Cases	Controls	SNPs	Cases	Controls	SNPs
**FR**	466	0	567,589	460	0	529,347	449	0	412,212
**NL**	286	1,637	574,856	269	1,560	423,769	264	1,559	412,212
**GER**	239	976	561,466	235	945	506,183	233	943	412,212
**USA**	280	808	476,964	262	796	452,558	232	750	412,212
**UK**	286	3,751	480,729	269	3,739	442,050	267	3,735	412,212
**Total**	**1,557**	**7,172**	**-**	**1,495**	**7,040**	**-**	**1,445**	**6,987**	**412,212**

The mean AAO for EOPD cases was 41.36 years (n = 1427, range: 7–50, standard deviation (SD): 7.24), with 940 individuals AAO≤45 years and 581 individuals AAO≤40 years (see Figure S1 for histogram of AAO distribution). Average chronological age of EOPD cases was 59.54 as of 2010 (range: 27–95, SD: 9.59, n = 853), and the average chronological age of controls was 53.95 (range: 21–101, SD: 8.67, n = 6973). 37.1% of cases and 52.4% of controls were female.

### Runs of homozygosity

Initial identification of ROHs was performed using PLINK v1.07 [24]. A window of 50 SNPs was defined as homozygous if it contained at most 1 heterozygous genotype and 1 missing genotype. Such windows were moved across the genome, and a SNP was counted as part of a ROH if >5% of windows spanning it were homozygous. These are the values suggested in PLINK, designed to minimize the probability of a window being called homozygous by chance, while ensuring that SNPs on the edge of a true ROH will be assigned to that ROH. Each ROH had to contain on average at least 1 SNP per 50 kb. The minimum length of the ROH was set at 1 Mb and sequentially increased by 1 Mb up to 10 Mb.

Plotting these ROHs as custom tracks in UCSC genome browser (http://genome.ucsc.edu/) showed that the majority of 1–2 Mb ROHs, occur in clusters containing hundreds to thousands of samples and are, most likely, identical by state (IBS) instead of identical by descent (IBD). A SNP locus or region of loci is said to be IBD if the homozygous alleles have originated from the same ancestor, while IBS refers to loci/regions that are merely homozygous by chance. In an attempt to remove homozygous runs that occurred at a high frequency in the sample population and that might bias our downstream analyses, we identified all regions at which at least 1% of all individuals (both cases and controls) harboured a ROH of 2 Mb or more in length. Within most of these regions, the mean length of overlapping common ROHs tended to be largely uniform. However some rare ROHs (<1% frequency in the study population) that were considerably longer and thus more likely to be IBD appeared to span some of these regions independently of the common ROHs. Those whose length deviated more than 3 SD from the mean length of the ROHs in that region, were retained in the analysis (figure S2 illustrates which ROHs would remain in the analysis following this approach in an example of one such region). This filtering approach was repeated for ROHs with a minimum length of 3, 4, and 5 Mb. At 6 Mb and above, no more regions with a ROH frequency >1% were identified.

### Statistical analyses

#### Basic homozygosity burden analysis

The proportion of individuals with at least one ROH of a given minimum length and the total number of ROHs per individual (rate) were calculated for both all ROH and rare ROH. Means were compared between cases and controls using simple one-tailed T-tests, and empirical p values were generated by permuting case/control status (100 million permutations, resulting in accuracy up to 1×10^−8^).

#### Homozygosity burden analysis using logistic regression models

In order to estimate the magnitude of risk associated with elevated levels of genome wide homozygosity, and to allow the inclusion of potentially confounding variables, analyses were further refined with a series of logistic regression models. Thus, the proportion of samples with ROHs above a given length and the total number of ROHs per individual were modelled separately as independent variables with case-control status as the dependent variable. Chronological age as of 2010 (when available) and the two first components (C1 and C2) of the multidimensional scaling matrix (MDS) (which captures about 90% of genetic variation in Caucasian populations - see supporting information S1) were included as covariates. To remove the possibility of chance occurrences of homozygosity affecting our statistical calculations, genome-wide rates of homozygosity (*f* coefficients) outside of linkage disequilibrium were calculated using PLINK v.1.07, in a linkage disequilibrium trimmed dataset (supporting information S1). This statistic summarizes the proportion of genotypes in our trimmed dataset that deviated from the expected number of homozygous genotypes in each population under assumptions of Hardy-Weinberg equilibrium. A more negative *f* would suggest a high level of heterozygosity in a sample; a more positive *f* estimate would suggest elevated rates of homozygosity beyond what is expected under normal assumptions. This statistic was then applied as a covariate to our logistic regression models. Logistic models were executed using R v.2.11.1 [25].

#### Exclusion of known PARK loci

In order to test whether the excess of homozygosity detected might be due to carriers of homozygous mutations in the most prominent *PARK* genes, both the one-tailed t-tests and the logistic regression models were repeated after excluding those samples with ROHs overlapping the genomic position of any of these genes. For this purpose, the start and end positions of RefSeq genes for *ATP13A2*, *FA2H*, *FBXO7*, *LRRK2*, *PARK2*, *PARK7*, *PINK1*, *PDXK*, *PLA2G6*, *SNCA*, and *SPG11*, were downloaded from the UCSC table browser (build NCBI36/hg18). If more than one transcript was present, the longest available transcript was used as a reference. A total of 47 cases and 184 controls harboured ROHs>2 Mb in length overlapping with at least one of these genes.

#### Homozygosity Mapping

Two approaches were used to find genomic regions in which extended homozygosity differed between cases and controls. First, we used PLINK v1.07 to define pools of overlapping ROHs. Each pool contained at least two different ROHs that did not have to match allelically. This approach identified 1,820 unique pools containing two or more ROHs. The genomic region spanned by all the runs in a certain pool was used to define 1,604 unique consensus regions spanning at least 2 consecutive SNPs. The number of times each of these consensus regions was completely overlapped by ROHs in cases and controls was counted and p values calculated based on 100,000 permutations. Multiple test corrections were applied based on the number of consensus regions tested.

In a second approach (gene-based) trying to identify genomic regions differentially overlapped by ROHs in cases versus controls in our cohort, a list containing the genomic coordinates of 19,058 genes and predicted transcripts in the human genome (NCBI B36 assembly) was downloaded from the PLINK resources website (http://pngu.mgh.harvard.edu/~purcell/plink/res.shtml). Using PLINK v1.07, the number of times a given gene was overlapped by ROHs in cases and controls was counted and p values were calculated based on 100,000 permutations. P values were multiple test-corrected based on the number of genes and predicted transcripts in our list.

## Results

A total of 216,660 homozygous runs ranging in size from 1 to ∼71.6 Mb (mean length: 1.4 Mb) and containing 50 to 9,743 contiguous homozygous SNPs, were identified. The exact number of ROHs identified at any given size threshold in cases and controls can be found in table S2. Every case and control had at least one ROH measuring more than 1 Mb (table S3 a). The mean number of ROH (greater than 1 Mb) per person was 25.5 (25.22 in cases, and 25.79 in controls, ratio: 0.98, p = 1, table S3 b). We therefore focused on ROH of at least 2 Mb length in subsequent analyses, of which there were 19,025 in our dataset.

### Basic homozygosity burden analysis

Around 88% of cases and 90% of controls harboured ROHs of at least 2 Mb length (ratio: 0.98, p = 0.97). However, at 3 Mb minimum length, a small but significant increase in the proportion of cases with ROHs versus controls became apparent (42.6% vs. 39.4%, ratio: 1.08, p = 0.01). The biggest difference in the proportion of samples with an ROH of a given minimum size was seen at 9 Mb (4.4% vs. 1.4%, ratio: 3.17, p<1×10^−8^) (table S3 a). At a minimum length of 2 Mb, the average number (rate) of ROHs in cases was 2.24 and 2.26 in controls (ratio: 0.99, p = 0.65). At a minimum length of 3 Mb, the rate in cases and controls dropped to 0.65 and 0.52, respectively, indicating a highly significant excess of homozygosity in cases (ratio: 1.25, p = 2.30×10^−6^). Again, the biggest difference was seen at a threshold of 9 Mb, with a rate of 0.08 in cases, and 0.02 in controls (ratio: 3.48, p<1×10^−8^) (table S3 b). This initial analysis showed a clear excess of homozygosity by all metrics tested in cases as compared to controls.

**Figure 1:**
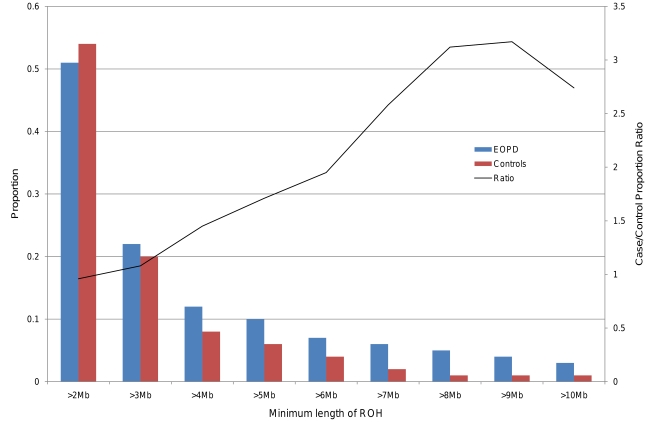
Number of rare ROHs at different size thresholds in EOPD and control groups. In this bar plot the average number of rare ROHs per person (rate) in either EOPD (red) or control (blue) groups is shown for different minimum size thresholds. The black line represents the ratio of average rate in cases vs. average rate in controls. Differences were statistically significant from a threshold of 2 Mb (0.91 vs. 0.82, ratio: 1.09, p = 0.01) and remained strongly significant throughout, peaking at 9 Mb (0.04 vs. 0.01, ratio: 3.17, p<1.00×10^−8^.

**Figure 2:**
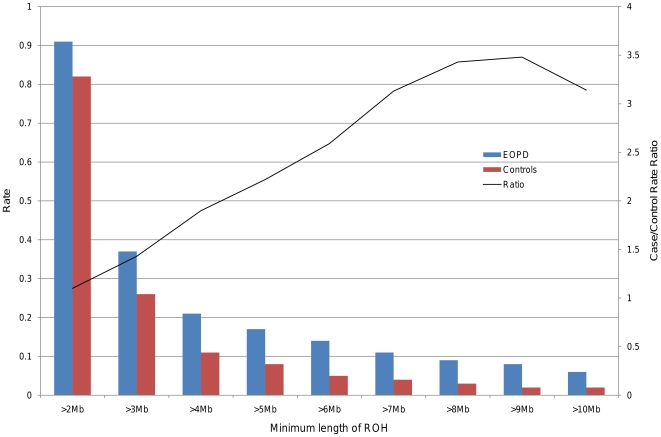
Proportion of cases and controls with rare ROH of a given minimum size. This bar plot displays the proportion of individuals presenting with at least one ROH of a given size threshold in EOPD (red) and control groups (blue). The ratio of the case/control proportions is represented by the black line. The difference between ROH-positive proportions in cases and controls became statistically significant at 4 Mb (0.12 vs. 0.08, ratio: 1.45, p = 4.30×10^−6^), and remained highly significant throughout higher size thresholds.

Analysis was repeated following filtering for rare ROHs. The number of rare ROHs is shown in table S2 b). When taking only rare ROHs into consideration, the case-specific excess of the number of homozygous runs per person (rate) became more pronounced, reaching statistical significance at a size threshold of 2 Mb (0.91 vs. 0.82, ratio: 1.10, p = 0.01) and remained strongly significant throughout (table 2 a, figure 1). The proportion of individuals with at least one rare ROH of a given length dropped overall, and the difference between cases and controls became significant at 4 Mb (0.12 vs. 0.08, ratio: 1.45, p = 6.50×10^−6^) (table 2 b, figure 2). As expected, there was no increased burden in cases when the analysis was performed using exclusively common ROHs with a frequency >1% (data not shown).

**Table 2:** Proportion and Rate of rare ROHs in EOPD cases and controls.

	a) Proportion				b) Rate			
Size	EOPD	Controls	Ratio	P value	EOPD	Controls	Ratio	P value
>2 Mb	0.51	0.54	0.96	0.94	0.91	0.82	1.10	0.01
>3 Mb	0.22	0.20	1.08	0.10	0.37	0.26	1.43	2.00×10^−06^
>4 Mb	0.12	0.08	1.45	6.50×10^−6^	0.21	0.11	1.90	1.00×10^−07^
>5 Mb	0.10	0.06	1.71	<1.00×10^−8^	0.17	0.08	2.22	<1.00×10^−08^
>6 Mb	0.07	0.04	1.95	<1.00×10^−8^	0.14	0.05	2.59	<1.00×10^−08^
>7 Mb	0.06	0.02	2.58	<1.00×10^−8^	0.11	0.04	3.13	<1.00×10^−08^
>8 Mb	0.05	0.01	3.12	<1.00×10^−8^	0.09	0.03	3.43	<1.00×10^−08^
>9 Mb	0.04	0.01	3.17	<1.00×10^−8^	0.08	0.02	3.48	<1.00×10^−08^
>10 Mb	0.03	0.01	2.74	1.30×10^−7^	0.06	0.02	3.14	4.78×10^−06^

a) Proportion of samples with at least one rare ROH of a given minimum size.

b) Rate of rare ROHs of a given minimum size.

### Homozygosity burden analysis using logistic regression models

Logistic models both without (Model 1) and with covariates (*f* coefficient, age, MDS factors) (Models 2–4) suggest a highly significant association between the presence of one or more particularly longer ROHs (proportion) and disease phenotype (Tables S4a, S5a), as well as the rate of ROHs and phenotype (tables S4 b, S5 b). Including the genome-wide rate of homozygosity outside of LD (i.e. *f*) as a covariate decreased the average log10(p) by 2.1 units (Model 2). In other words, controlling for background genome wide homozygosity increased the detection of differences in ROHs between cases and controls. Taking into account chronological age increased the average log10(p) by 3.9, suggesting that the marginally older age of our cases had a confounding effect (Model 3). Accounting for potential population stratification had an impact on the significance of our findings, also increasing the average log10(p) by 3.86, (Model 4, table 3). The measure of rate is more attenuated through incorporation of all covariates than the measure of proportion. However, rate as well as proportion measures still remained significantly associated with case status from a minimum ROH length of 3 Mb onwards.

**Table 3:** Logistic models for proportion and rate of rare ROHs.

	a) Proportion		b) Rate	
Size	*P value*	*Odds Ratio (95%CI)*	*P value*	*Odds Ratio (95%CI)*
>2 Mb	n.s.	1.00 (0.86–1.16)	n.s.	1.04 (0.99–1.10)
>3 Mb	0.02	1.22 (1.02–1.45)	3.08×10^−3^	1.12 (1.04–1.22)
>4 Mb	7.31×10^−5^	1.57 (1.25–1.96)	0.01	1.14 (1.03–1.26)
>5 Mb	8.26×10^−6^	1.77 (1.37–2.26)	0.01	1.17 (1.04–1.31)
>6 Mb	1.19×10^−5^	1.91 (1.42–2.53)	4.72×10^−3^	1.21 (1.06–1.38)
>7 Mb	3.25×10^−7^	2.39 (1.70–3.31)	0.01	1.22 (1.06–1.42)
>8 Mb	7.73×10^−7^	2.59 (1.76–3.74)	0.01	1.23 (1.05–1.46)
>9 Mb	2.25×10^−6^	2.62 (1.74–3.88)	0.01	1.27 (1.06–1.54)
>10 Mb	1.35×10^−3^	2.09 (1.32–3.25)	n.s.	1.21 (0.98–1.49)

a) Logistic model 4 (adjusted for *f*, chronological age, and MDS covariates) with phenotype (EOPD case or control, 1 or 0) as dependent variable and proportion of samples with at least one rare ROH of a given minimum size as independent variable.

b) Logistic model 4 (adjusted for *f*, chronological age, and MDS covariates) with phenotype (EOPD case or control, 1 or 0) as dependent variable and rate of rare ROHs per person of a given minimum size as independent variable. (Models 1–3 in supplementary material).

### Investigating the source of excess homozygosity in EOPD

Given that the most striking differences were apparent at ROHs of 8–9 Mb in length, we further investigated the role of individuals with ROHs of >8 Mb (71 cases and 110 controls). Removing these individuals from the analysis led to a complete loss of significant differences in both the proportion of samples with ROHs (table S6 a) and the rate of ROHs per person (table S6 b). While a degree of significance was lost due to the exclusion of rare long ROHs of 8 Mb and above, these individuals also carried an excess of shorter ROHs. Differences in proportion (table S7 a) and rate (table S7 b) were still significant after restricting analysis to only ROHs below 8 Mb in length. We observed that those 71 cases that had at least one ROH>8 Mb had a higher frequency of runs between 2–7 Mb than the remaining 1,374 cases (3.78 vs 2.07, ratio: 1.83, p<1×10^−8^; figure S3). The average AAO of these 71 cases was 40.73 years (range: 13–50, SD: 8.23, n = 70), and not significantly different from the rest of cases (AAO: 41.25, SD: 7.21, range: 7–50, n = 1351) as assessed by two-sample two-tailed t-test assuming equal variances (p = 0.56). There was a 10-fold increase of the mean *f* coefficient when comparing samples with or without 8 Mb ROHs (0.014 versus 0.001, respectively, p = 1.16×10^−16^). The distribution of cases with 8 Mb ROH across the different populations under study was as follows: 25.35% France, 25.35% Germany, 23.94% Netherlands, 15.49% USA and 9.86% UK. This did not differ significantly from the distribution of cases without 8 Mb ROH, apart from the observation that German cases were more likely to have 8 Mb ROH than any other (Fisher's exact test p value = 0.01). 7 of the 71 individuals (9.86%) carried a ROH of >2 Mb across *PARK2* (parkin), *ATP13A2*, *FBXO7* or *PLAS2G6*, which was a significantly higher percentage of carriers than that found in cases without long ROH (2.91%, p = 0.01).

### Homozygosity Mapping

The first homozygosity mapping approach involved testing consensus regions where two or more ROHs overlapped at a minimum of 2 SNPs for association with disease phenotype. In the present dataset there were 1,604 consensus regions overlapped by rare ROHs of at least 2 Mb length. One consensus region, located on chromosome 19p13.3, remained significantly associated with EOPD after correction for multiple testing (uncorrected p value = 4.00×10^−5^, corrected p = 5.79×10^−3^, Consensus #1 in table 4). 6 cases and no controls carried a ROH spanning this region. These cases were originally from Germany (3 cases) and the UK, France and USA (1 case each). The mean AAO in these cases was 39 (range = 29–49) and not significantly different from other cases as shown by a two tailed t-test (p = 0.54). Interestingly all six cases belonged to the group of cases with at least one ROH above 8 Mb length, although the longest ROH for a given person was not necessarily overlapping consensus region #1 (mean length of ROH = 7.5 Mb; range = 2.9–16.3 Mb).

**Table 4:** Top 10 associated consensus regions.

Consensus	Chr.	Start-End	EOPD	Controls	P	P*
#1	19	6,313,724–6,498,141	6(0.42%)	0(0.00%)	4.00×10^−5^	5.79×10^−3^
#2	3	7,263,365–7,356,271	4(0.28%)	0(0.00%)	8.90×10^−4^	0.17
#3	9	18,914,463–20,149,208	4(0.28%)	0(0.00%)	9.40×10^−4^	0.17
#4	4	57,197,834–57,284,828	4(0.28%)	0(0.00%)	9.90×10^−4^	0.17
#5	10	81,888,175–82,416,665	5(0.35%)	1(0.01%)	7.00×10^−4^	0.26
#6	4	58,379,742–58,393,395	7(0.48%)	4(0.06%)	5.30×10^−4^	0.33
#7	19	10,332,977–12,341,037	5(0.35%)	2(0.03%)	2.10×10^−3^	0.51
#8	19	3,747,849–4,335,674	5(0.35%)	2(0.03%)	2.29×10^−3^	0.51
#9	5	56,371,946–56,626,551	5(0.35%)	2(0.03%)	2.39×10^−3^	0.51
#10	5	54,228,580–54,301,303	5(0.35%)	2(0.03%)	2.45×10^−3^	0.51

P = uncorrected p value.

P* = p value corrected for multiple testing using 100,000 case/control status permutations.

This consensus region spans ∼184 kb in chromosome 19p13.3 and contains 12 genes and predicted transcripts, namely: *CLPP*, *ALKBH7*, *PSPN*, *GTF2F1*, *KHSRP*, *MIR3940*, *SLC25A41*, *SLC25A23*, *CRB3*, *DENND1C*, *TUBB4* and *TNFSF9* (figure S4). The presence of deletions and duplications in this region was excluded by visual examination of the genotyping intensity data (data not shown). There was no extended shared haplotype among cases with homozygosity in the 19p13.3 region. Sanger sequencing of all exons and exon-intron boundaries of genes and predicted transcripts contained in this genomic region failed to find any associated variants. Another 118 consensus regions were nominally associated with EOPD (p<0.05). However, none of them passed correction for multiple testing. A list of the top 10 associated regions can be found in table 4.

In the second approach of homozygosity mapping, all genes and predicted transcripts according to NCBI B36 assembly were used as the unit of analysis. Of the 19,058 genes queried, a total of 17,182 were spanned by at least one ROH. Eleven genes across a 174 kb stretch on chromosome 19 were intersected by significantly more ROHs in the cases compared to controls, a finding that remained significant following multiple test correction (p = 0.01) and was mostly overlapping with the region identified by the consensus approach described above.

Nominal significance was also achieved for an additional 1,816 genes; however these did not withstand genome wide correction. Given that a single ROH will typically span multiple genes, then in the context of this experiment each locus is not truly independent and therefore the genome wide correction for ∼19,000 independent tests is highly conservative. This is emphasised by *post hoc* analysis which revealed that, as expected, a large proportion of the associated genes were spanned by the same set of ROHs and it was possible to assign all 1,827 genes to 300 independent groups containing 1 – 66 genes each (table 5 shows the top 10 regions with uncorrected p values≤0.01; figure S5 shows the three most significantly associated gene groups on chromosome 19p13.3).

**Table 5:** Top 10 associated gene groups.

Group	Chr.	Start	End	EOPD	Controls	P	P*	Genes
#1	19	6312462	6486933	6(0.42%)	0(0.00%)	3.00×10^−5^	0.01	CLPP, ALKBH7, PSPN, GTF2F1. KHSRP. SLC25A41. SLC25A23, CRB3, DENND1C, TUBB4, TNFSF9
#2	10	80591875	81842287	5(0.35%)	0	1.10×10^−4^	0.07	ZMIZ1, PPIF, ZCCHC24, EIF5AL1, SFTPA2, SFTPA2B, SFTPA1B, SFTPA1, SFTPA2, SFTPA2B, SFTPA1B, SFTPA1, SFTPD, C10orf57
#3	19	5774817	6284562	5	0	1.30×10^−4^	0.07	NRTN, FUT6, FUT3, FUT5, NDUFA11, VMAC, CAPS, RANBP3, RFX2, ACSBG2, MLLT1, ASAH3
#4	19	6536849	6702529	5	0	1.50×10^−4^	0.07	CD70, TNFSF14, C3, GPR108, TRIP10
#5	5	70787197	71052628	4(0.28%)	0	6.90×10^−4^	0.34	BDP1, MCCC2, CARTPT
#6	4	114593020	114902177	5	1(0.01%)	7.00×10^−4^	0.45	CAMK2D
#7	19	4353659	5742190	5	1	7.10×10^−4^	0.45	CHAF1A, UBXD1, HDGF2, KIAA1881, LSDP5, LRG1, SEMA6B, TNFAIP8L1, C19orf10, DPP9, FEM1A, TICAM1, M6PRBP1, ARRDC5, UHRF1, JMJD2B, PTPRS, ZNRF4, SAFB2, SAFB, P117, HSD11B1L, RPL36, LONP1, TMEM146, MGC24975, DUS3L
#8	10	81882237	82396296	5	1	7.30×10^−4^	0.45	PLAC9, ANXA11, MAT1A, DYDC1, DYDC2, C10orf58, TSPAN14, SH2D4B
#9	4	55907165	56197222	4	0	7.50×10^−4^	0.34	SRD5A3, TMEM165, CLOCK, PDCL2, NMU
#10	15	64781687	64861380	7(0.48%)	4(0.06%)	7.50×10^−4^	0.51	SMAD6

P = uncorrected p value.

P* = p value corrected for multiple testing using 100,000 case/control status permutations.

### Exclusion of known *PARK* loci

All samples with ROHs of above 2 Mb overlapping any of the 12 known parkinsonism and pallido-pyramidal syndrome genes were removed. After removal, both the basic burden analysis and logistic regressions models produced similar results as those described above (tables S8, S9, S10, and S11). These results indicate that excess of homozygosity outside the known parkinsonism and pallido-pyramidal syndrome genes represent a risk for EOPD in our population and that further recessive genes are yet to be identified. Performing the gene-based homozygosity mapping explained before in the aforementioned genes, resulted in the association displayed in table S12. To note, only *PARK2* (parkin) was statistically more often overlapped by ROHs in cases versus controls after multiple test correction (p = 0.04), further supporting the idea that novel genetic factors are underlying the excess of homozygosity detected in our cases. Additionally, any evidence of association of ROH across *PARK2* (parkin) disappeared when 2 cases with known mutations in the gene were excluded from the analysis (p = 0.31, p corrected = 0.86).

## Discussion

In this work, we have demonstrated an increased rate of genomic homozygosity based on an increased proportion of EOPD patients with long homozygous sequences as compared to controls. In addition, two different homozygosity mapping approaches identified a region in chromosome 19p13.3 in which cases carried significantly more ROHs than controls.

This study was based on a very large series of patients with EOPD. The excess number of ROHs in cases becomes apparent at a threshold of 3 Mb for all homozygous tracts and at 2 Mb when considering only rare homozygous tracts. The effects became most pronounced at a threshold of 9 Mb where there is a 3.5 fold increase in the proportion of EOPD cases with a homozygous track as compared to controls.

Possible confounding effects in this work include ethnic/regional and age differences between cases and controls and the retention of cases with mutations in known genes. Because differences in homozygosity measures among Caucasian populations have been reported previously [21], [26], C1 and C2 components of the population MDS matrix were included as co-factors in our analyses. This approach attenuated statistically significant differences in the rate of ROH (p = 0.01), but did not affect the highly significant differences in proportions.

Age factors were addressed by ensuring approximate matching in the ages of cases as compared to controls (average year of birth 1950 vs. 1956, respectively) and by carrying out regression analyses including chronological age as a covariate. Besides, inbreeding coefficients (*f*) calculated on a LD-pruned version of our dataset were included as covariates in our models to correct for autozygosity differences across different generations in outbred individuals [27]. The effect of this coefficient merits particular comment. Including the *f* as a covariate increased case-control differences suggesting that the excess of homozygous runs in cases does not relate to genome wide homozygosity, but rather to an excess of a small number of longer runs of homozygosity, likely to be homozygous by descent and to contain pathogenic mutations. In controls, the proportion and rate of homozygous runs relates more directly to the effect of background genome wide homozygosity.

Furthermore, the effect of occult homozygous mutations in genes previously associated with EOPD or other related disorders was excluded by repeating the analysis without those samples with ROHs overlapping known loci for PD, pallido-pyramidal and parkinsonism dystonia genes. Of note, gene-based homozygosity analysis in these genes revealed that only ROHs overlapping *PARK2* (parkin) were associated with EOPD after correcting for multiple tests. Removal of the samples involved in this association did not remove the association described after our basic burden and logistic regression analyses. These results strongly suggest the role of other unknown genetic factors playing an important role in the risk for recessive EOPD.

In other disorders, similar analyses aimed at showing an increased homozygous burden in cases have resulted in mixed results. One study reported an increased homozygous burden in patients with colorectal cancer [28] but this finding was not replicated [29]. In a study of ROH in bipolar disorder, no increased burden was seen [30]. Lencz and colleagues [31] demonstrated the presence of 9 ROHs significantly overrepresented in a cohort of 178 patients with schizophrenia as compared to 144 healthy controls. Hildebrandt and colleagues [22] demonstrated the feasibility of homozygosity mapping using SNP microarrays by investigating ROH in individuals from families with varying degrees of consanguinity and two different paediatric autosomal recessive kidney diseases, and 93% of known mutations were identified in ROH of sizes as small as 2 Mb. In the realm of neurological disorders, one study investigated homozygous runs in ostensibly outbred individuals with late onset AD and identified a trend to excess homozygosity in AD, as well as one consensus region spanning 7 genes, which was significantly more common in cases as compared to controls [32], however this finding was not replicated in a more recent study [33].

Finally, using two different homozygosity mapping approaches one region on the short arm of chromosome 19 was found to be associated with EOPD after correcting for multiple testing. Six different cases from four different countries contained rare ROHs overlapping in a ∼184 kb stretch in this chromosome 19. Twelve genes were contained within the region. Of particular interest is *PSPN*, which encodes persephin, a neurotrophic factor shown to promote survival of ventral midbrain dopaminergic neurons in vitro [34]. Visual examination of the genotyping intensity data of the samples involved in this association failed to find any structural variant overlapping with this genomic region (data not shown). Also, exome sequencing of these samples failed to find any variant to be associated with EOPD in our population (data not shown). These results might suggest that a non-coding variant or a more complex structural alteration is leading to disease in these patients. Sequence capture of the entire region may help understanding the etiology of EOPD in these cases.

We succeeded in showing an excess of homozygosity (in terms of ROHs) in EOPD cases versus controls. To note, these ROHs are not necessarily harbouring disease-causing variants. However, a small proportion of them might carry recessive alleles associated with EOPD. Although we succeeded in identifying one candidate region using homozygosity mapping in our cohort, this related to a small number of cases and a new Mendelian gene was not identified. There are a number of possible factors which may explain our results: i) a larger population is needed to detect new genes for EOPD in unrelated cases, ii) there may be common pathogenic mutations in shorter ROHs in inbred individuals which are difficult to detect through conventional mapping, iii) there may be a large number of recessive genes for EOPD which are highly penetrant but individually uncommon, or iv) the effect is explained by a burden of multiple low penetrance homozygous alleles in cases.

Further work in which ROH are analysed with whole exome data will be needed to resolve these issues. The identification of autosomal recessive genes for EOPD to date has been based on the identification of specific consanguineous families. Novel genetic technologies will allow us to determine new Mendelian genetic factors, without traditional linkage and positional cloning. The present work indicates our current knowledge of the genetic aetiology of EOPD is incomplete and that given sufficient sample size it should be possible to clone new autosomal recessive genes for EOPD following the investigation of apparently outbred unrelated patients.

## Supporting Information

Information S1 **Quality control methods.**
(DOC)

Figure S1 **Distribution of AAO in the case population under study.**
(TIF)

Figure S2 **Histogram depicting frequency of ROH of given lengths in a region containing common ROH.** According to our filtering approach, only the ROH in red would remain in the analysis on the basis of their length exceeding 3× SD+Mean. In this example: 3592.39 kb+3098.77 kb = 6691.16 kb
(TIF)

Figure S3 **Frequency of ROHs in cases with and without a ROH of >8 Mb length.** 71 cases were found to harbour at least one ROH of at least 8Mb length. Comparing these individuals against the remainder of cases (n = 1374) shows a small but significant rise in the number of ROH in those 71 cases, at various ROH lengths.
(TIF)

Figure S4 **Consensus associated region in chromosome 19p13.3.** ROHs in cases are shown in red. Consensus region to all ROHs in the region is shown in blue. No ROHs in controls spanned this region. RefSeq genes and transcripts in region are shown in blue.
(TIFF)

Figure S5 **Three most significantly associated gene groups on chromosome 19.** Genes spanned by ROHs in cases significantly more often than by ROHs in controls are shown in red. The three associated gene groups are shown in black. Blue bars denote case ROH, and brown bars denote control ROH. White arrows signify that a ROH continues beyond the borders of the image. The scale bar is 1Mb long.
(TIFF)

Table S1 **Number of samples excluded during QC.** a) Number of samples excluded during QC (1). b) Number of samples excluded during QC (2)
(DOC)

Table S2 **ROH metrics.**
(DOC)

Table S3 **Burden analysis.** a) Proportion of samples with ROH of a given minimum size. b) Rate of ROH of a given minimum size
(DOC)

Table S4 **Logistic models.** a) Logistic models with proportion of samples with at least one ROH of a given minimum size as independent variable, and phenotype as dependent variable. b) Logistic models with rate of ROH of a given minimum size as independent variable, and phenotype as dependent variable. (Covariates - Model 1: unadjusted; Model 2: *f*; Model 3: *f*, age; Model 4: *f*, age, MDS)
(DOC)

Table S5 **Logistic models.** a) Logistic models with proportion of samples with at least one rare ROH of a given minimum size as independent variable, and phenotype as dependent variable. b) Logistic models with rate of rare ROH of a given minimum size as independent variable, and phenotype as dependent variable. (Covariates - Model 1: unadjusted; Model 2: *f*; Model 3: *f*, age; Model 4: *f*, age, MDS)
(DOC)

Table S6 **Burden analysis following the exclusion of samples with ROH>8 Mb in size.** a) Proportion of samples with ROH of a given minimum size. b) Rate of ROH of a given minimum size.
(DOC)

Table S7 **Burden analysis following the exclusion of ROH>8 Mb in size.** a) Proportion of samples with ROH of a given minimum size. b) Rate of ROH of a given minimum size.
(DOC)

Table S8 **Burden analysis following the exclusion of samples with all ROH>2 Mb size across known** ***PARK*** **loci.** a) Proportion of samples with all ROH of a given minimum size. b) Rate of all ROH of a given minimum size.
(DOC)

Table S9 **Burden analysis following the exclusion of samples with rare ROH>2 Mb size across known** ***PARK*** **loci.** a) Proportion of samples with rare ROH of a given minimum size. b) Rate of rare ROH of a given minimum size.
(DOC)

Table S10 **Logistic models following the exclusion of samples with all ROH>2 Mb across known** ***PARK*** **loci.** a) Logistic models with proportion of samples with at least one ROH of a given minimum size as independent variable, and phenotype as dependent variable. b) Logistic models with rate of all ROH of a given minimum size as independent variable, and phenotype as dependent variable. (Covariates - Model 1: unadjusted; Model 2: *f*; Model 3: *f*, age; Model 4: *f*, age, MDS)
(DOC)

Table S11 **Logistic models following the exclusion of samples with rare ROH>2 Mb across known** ***PARK*** **loci.** a) Logistic models with proportion of samples with at least one rare ROH of a given minimum size as independent variable, and phenotype as dependent variable. b) Logistic models with rate of rare ROH of a given minimum size as independent variable, and phenotype as dependent variable. (Covariates - Model 1: unadjusted; Model 2: *f*; Model 3: *f*, age; Model 4: *f*, age, MDS)
(DOC)

Table S12 **Gene-based homozygosity mapping results of the most prominent PARK genes.** P = uncorrected p value. P* = p value corrected for multiple testing using 100,000 case/control status permutations
(DOC)
