# Supplementary material for: Cooperative Genome-Wide Analysis Shows Increased Homozygosity in Early Onset Parkinson's Disease
Source: PLoS One. 2012 Mar 12;7(3):e28787. doi: 10.1371/journal.pone.0028787 (PMC3299635; doi:10.1371/journal.pone.0028787)
Supplement: Information S1 — Quality control methods. (DOC) [file pone.0028787.s001.doc]

**SUPPORTING INFORMATION S1**

**Quality Control**

The first round of QC was performed on each sample population separately. Mitochondrial markers and SNPs on sex chromosomes were excluded. Markers with a call rate below 98%, minor allele frequency below 1% or with extreme departure from HWE (p < 1 x 10^-7^) in controls were excluded. SNPs that were found to be missing more often (CHI^2^ test, p < 1 x 10^-4^) in either cases or controls were excluded. Individuals were initially removed from analysis if they presented with mismatching sex information or a SNP call rate below 95%. Two control samples with excess heterozygosity (co-efficient of inbreeding (*f*, determined with PLINK) 4 standard deviations below mean) was excluded. One highly inbred control individual (*f* > 0.1) was also excluded from analysis. One case was removed due to a chromosomal abnormality on chromosome 1. Cryptic relatedness and ethnically mismatched individuals were identified using pair wise Identity By State (IBS) distances and Multidimensional scaling (MDS). Individuals within any given data set, which deviated more than four SD from the mean for the first and second dimension (which together capture ~90% genetic variability in Caucasian populations), were removed from further analysis. The pair wise clustering based on IBS distances is useful for making estimations of pair wise Identity by Descent (IBD) to find pairs of individuals who appear to be cryptically related in a random sample. By estimating the probability of sharing 0, 1, or 2 alleles IBD for any two individuals, a proportion of IBD can be calculated (PI-HAT = P [IBD = 2] + 0.5 x P [IBD = 1]). Using 0.125 as a threshold for PI-HAT, 110 sample pairs were considered to be closely related. Thus, one member of each pair was removed from further analysis, preferentially removing samples that occurred in more than one pair or were controls. Additionally, PI-HAT data was used to reveal potential sample duplicates within our datasets.

A second round of QC was performed upon merging the different country datasets together. The dataset was restricted to SNPs passing QC in all individual datasets. SNPs that exhibited significant (p < 1 x 10^-4^) differences in call rate parameters between case and control samples were excluded from analysis. All samples with a call rate below 95% were excluded from analysis. The proportion of IBD (see above) was recalculated for all pairs in the entire sample, and sample pairs were considered to be cryptically related if they shared an estimated 0.125 IBD, and one sample of each such a pair was removed. Finally, after recalculating the IBS distribution for the remaining samples along with 30 trios from Yoruba (Nigeria), 30 trios from the Tokyo area in Japan and 30 US-resident trios with Northern and Western European ancestry from the Centre d’Etude du Polymorphisme Humain (CEPH, Paris, France); data downloaded from the HapMap website, all individuals that deviated more than 4 SD from the mean for MDS dimensions 1 and 2 were removed from analysis.
