# Supplementary figures and images for: Cooperative Genome-Wide Analysis Shows Increased Homozygosity in Early Onset Parkinson's Disease
Source: PLoS One. 2012 Mar 12;7(3):e28787. doi: 10.1371/journal.pone.0028787 (PMC3299635; doi:10.1371/journal.pone.0028787)

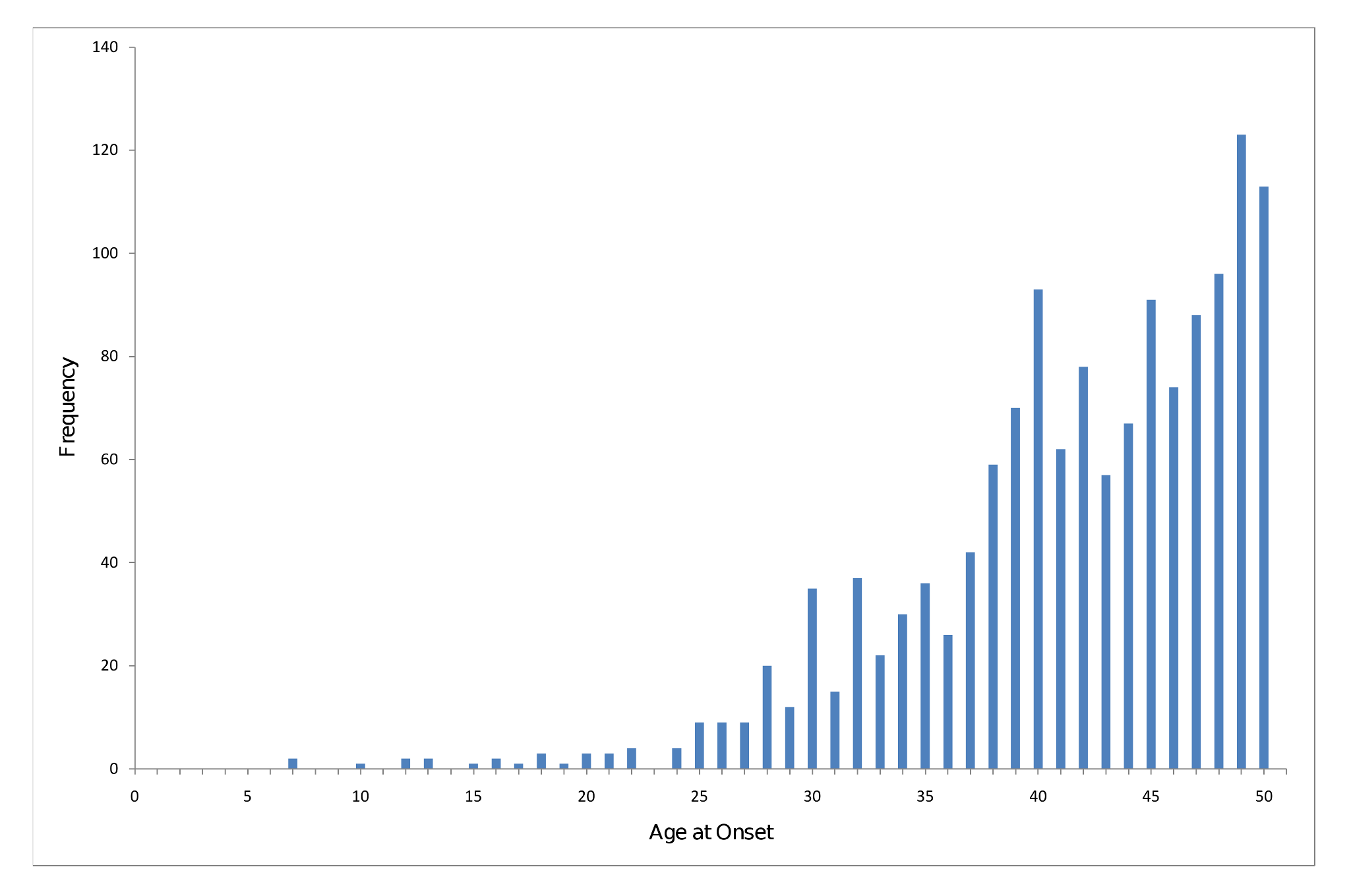

Supplement: Figure S1 — Distribution of AAO in the case population under study. (TIF) [file pone.0028787.s002.tif]

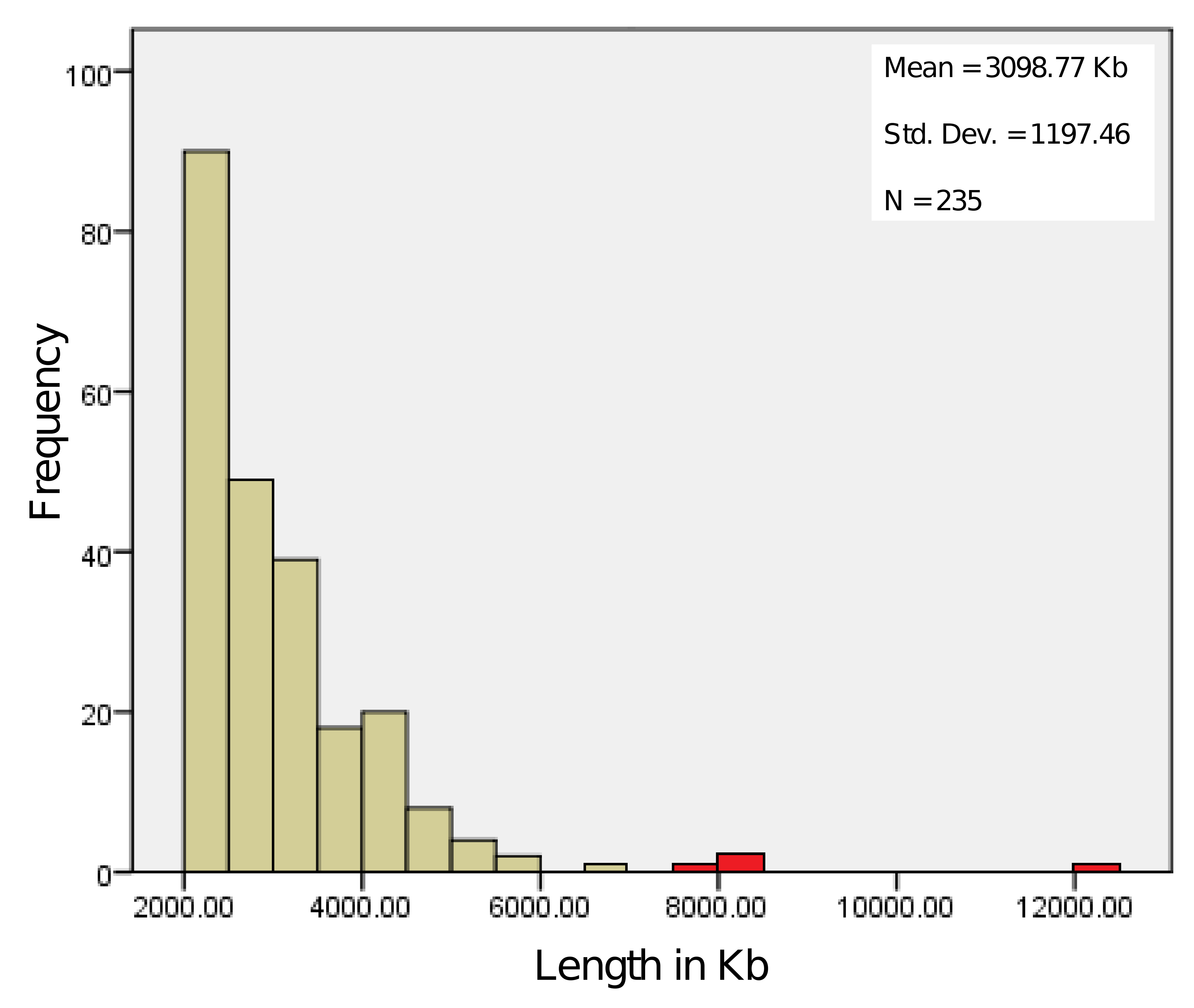

Supplement: Figure S2 — Histogram depicting frequency of ROH of given lengths in a region containing common ROH. According to our filtering approach, only the ROH in red would remain in the analysis on the basis of their length exceeding 3× SD+Mean. In this example: 3592.39 kb+3098.77 kb = 6691.16 kb (TIF) [file pone.0028787.s003.tif]

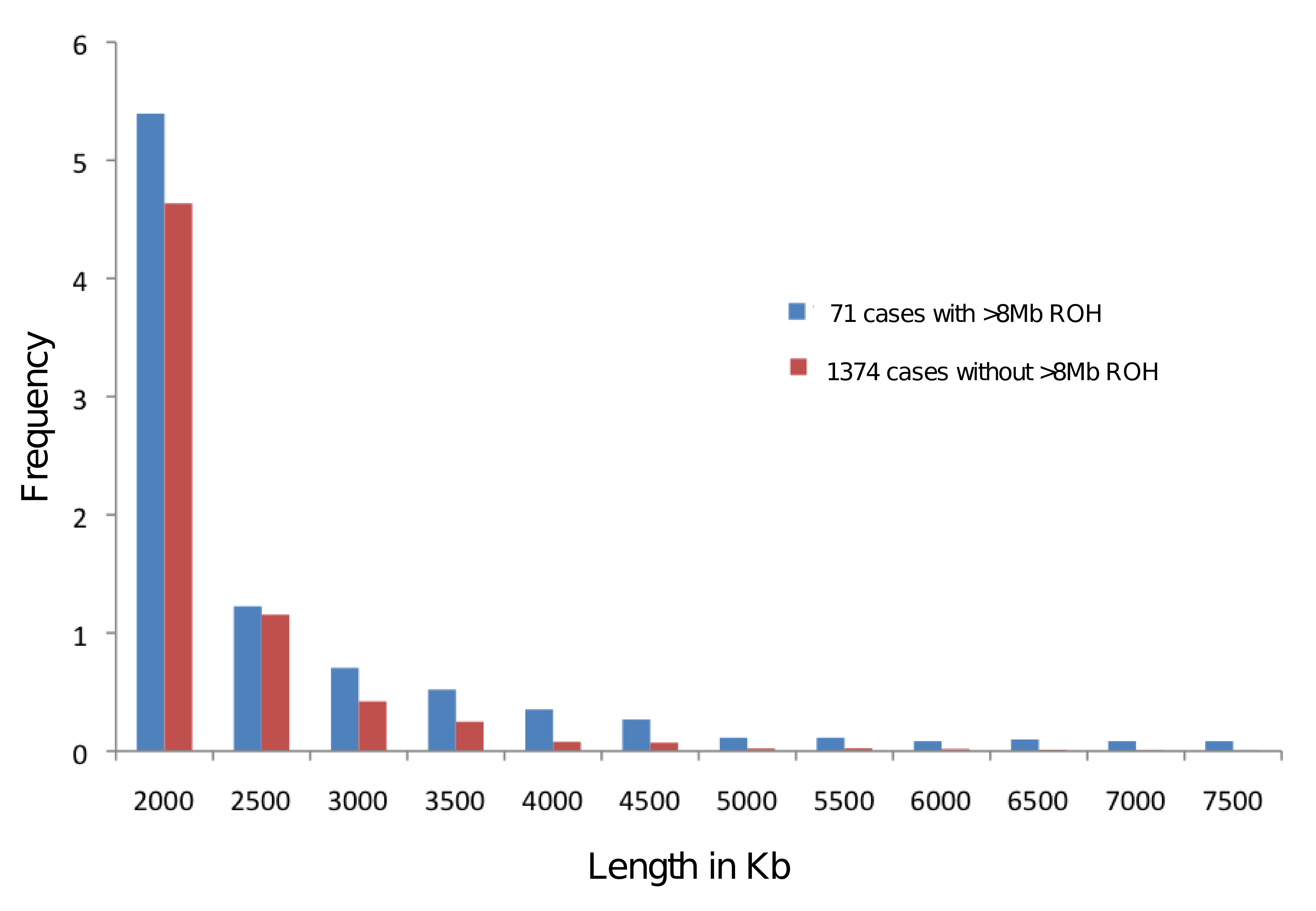

Supplement: Figure S3 — Frequency of ROHs in cases with and without a ROH of >8 Mb length. 71 cases were found to harbour at least one ROH of at least 8Mb length. Comparing these individuals against the remainder of cases (n = 1374) shows a small but significant rise in the number of ROH in those 71 cases, at various ROH lengths. (TIF) [file pone.0028787.s004.tif]

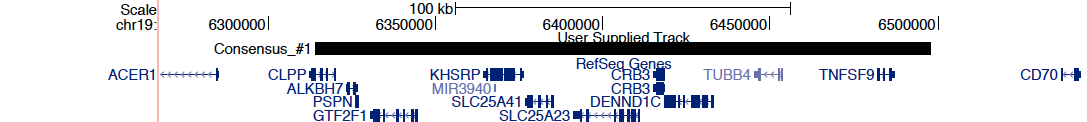

Supplement: Figure S4 — Consensus associated region in chromosome 19p13.3. ROHs in cases are shown in red. Consensus region to all ROHs in the region is shown in blue. No ROHs in controls spanned this region. RefSeq genes and transcripts in region are shown in blue. (TIFF) [file pone.0028787.s005.tiff]

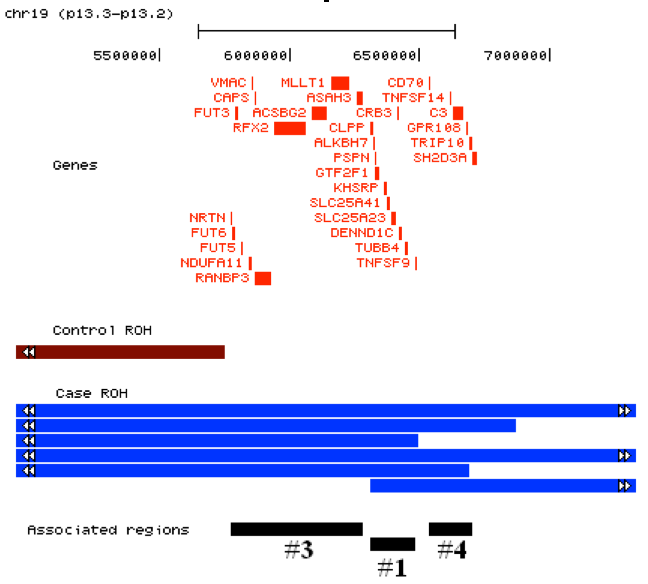

Supplement: Figure S5 — Three most significantly associated gene groups on chromosome 19. Genes spanned by ROHs in cases significantly more often than by ROHs in controls are shown in red. The three associated gene groups are shown in black. Blue bars denote case ROH, and brown bars denote control ROH. White arrows signify that a ROH continues beyond the borders of the image. The scale bar is 1Mb long. (TIFF) [file pone.0028787.s006.tiff]
