# Supplementary material for: Cooperative Genome-Wide Analysis Shows Increased Homozygosity in Early Onset Parkinson's Disease
Source: PLoS One. 2012 Mar 12;7(3):e28787. doi: 10.1371/journal.pone.0028787 (PMC3299635; doi:10.1371/journal.pone.0028787)
Supplement: Table S1 — Number of samples excluded during QC. a) Number of samples excluded during QC (1). b) Number of samples excluded during QC (2) (DOC) [file pone.0028787.s007.doc]

| **a) Reason for exclusion:** | **UK** | | **US** | | **German** | | **Dutch** | | **French** |
| --- | --- | --- | --- | --- | --- | --- | --- | --- | --- |
|  | **Cases** | **Controls** | **Cases** | **Controls** | **Cases** | **Controls** | **Cases** | **Controls** | **Cases** |
| **Low Call Rate** | 0 | 0 | 0 | 1 | 2 | 11 | 0 | 0 | 0 |
| **Low F / Contaminated** | 1 | 0 | 0 | 2 | 0 | 6 | 0 | 0 | 0 |
| **High F** | 0 | 0 | 0 | 1 | 0 | 0 | 0 | 0 | 0 |
| **MDS Outliers** | 5 | 11 | 14 | 2 | 2 | 4 | 7 | 0 | 5 |
| **Sex mismatch** | 0 | 0 | 0 | 0 | 0 | 0 | 0 | 0 | 0 |
| **Duplicates** | 9 | 0 | 0 | 0 | 0 | 0 | 0 | 0 | 0 |
| **Hidden Relatedness** | 2 | 1 | 4 | 6 | 0 | 10 | 10 | 77 | 0 |
| **Chromosomal Abnormality** | 0 | 0 | 0 | 0 | 0 | 0 | 0 | 0 | 1 |
| **Total** | *17* | *12* | *18* | *12* | *4* | *31* | *17* | *77* | *6* |

| **b) Reason for exclusion:** | **UK** | | **US** | | **German** | | **Dutch** | | **French** |
| --- | --- | --- | --- | --- | --- | --- | --- | --- | --- |
|  | **Cases** | **Controls** | **Cases** | **Controls** | **Cases** | **Controls** | **Cases** | **Controls** | **Cases** |
| **MDS Outliers** | 2 | 4 | 30 | 46 | 2 | 2 | 5 | 0 | 11 |
| **Hidden Relatedness** | 0 | 0 | 0 | 0 | 0 | 0 | 0 | 1 | 0 |
| **Total** | *2* | *4* | *30* | *46* | *2* | *2* | *5* | *1* | *11* |
