# Supplementary material for: Cooperative Genome-Wide Analysis Shows Increased Homozygosity in Early Onset Parkinson's Disease
Source: PLoS One. 2012 Mar 12;7(3):e28787. doi: 10.1371/journal.pone.0028787 (PMC3299635; doi:10.1371/journal.pone.0028787)
Supplement: Table S2 — ROH metrics. (DOC) [file pone.0028787.s008.doc]

|  | Number of ROH | | | Number of rare ROH | | |
| --- | --- | --- | --- | --- | --- | --- |
| ROH length | *Cases (n = 1445)* | *Controls (n = 6987)* | *Total* | *Cases* | *Controls* | *Total* |
| > 1Mb | 36,449 | 180,211 | 216,660 | na | na | na |
| > 2Mb | 3,239 | 15,786 | 19,025 | 1,309 | 5,761 | 7,070 |
| > 3Mb | 937 | 3,639 | 4,576 | 532 | 1,802 | 2,334 |
| > 4Mb | 426 | 1,356 | 1,782 | 310 | 788 | 1,098 |
| > 5Mb | 272 | 646 | 918 | 249 | 543 | 792 |
| > 6Mb | 201 | 375 | 576 | 201 | 375 | 576 |
| > 7Mb | 163 | 252 | 415 | 163 | 252 | 415 |
| > 8Mb | 134 | 189 | 323 | 134 | 189 | 323 |
| > 9Mb | 115 | 160 | 275 | 115 | 160 | 275 |
| > 10Mb | 87 | 134 | 221 | 87 | 134 | 221 |
