# Supplementary material for: Cooperative Genome-Wide Analysis Shows Increased Homozygosity in Early Onset Parkinson's Disease
Source: PLoS One. 2012 Mar 12;7(3):e28787. doi: 10.1371/journal.pone.0028787 (PMC3299635; doi:10.1371/journal.pone.0028787)
Supplement: Table S3 — Burden analysis. a) Proportion of samples with ROH of a given minimum size. b) Rate of ROH of a given minimum size (DOC) [file pone.0028787.s009.doc]

|  | 1. Proportion | | | | 1. Rate | | | |
| --- | --- | --- | --- | --- | --- | --- | --- | --- |
| **Size** | **EOPD** | **Controls** | **Ratio** | **P value** | **EOPD** | **Controls** | **Ratio** | **P value** |
| >1Mb | 1 | 1 | 1 | 1 | 25.22 | 25.79 | 0.98 | 1 |
| >2Mb | 0.88 | 0.90 | 0.98 | 0.97 | 2.24 | 2.26 | 0.99 | 0.65 |
| >3Mb | 0.43 | 0.39 | 1.08 | 0.01 | 0.65 | 0.52 | 1.25 | 2.30 x 10^-6^ |
| >4Mb | 0.19 | 0.16 | 1.19 | 3.00x 10^-3^ | 0.30 | 0.19 | 1.52 | 8.00 x 10^-7^ |
| >5Mb | 0.11 | 0.07 | 1.58 | 4.00 x 10^-7^ | 0.19 | 0.09 | 2.04 | <1.00 x 10^-7^ |
| >6Mb | 0.07 | 0.04 | 1.95 | <1.00 x 10^-7^ | 0.14 | 0.05 | 2.59 | <1.00 x 10^-7^ |
| >7Mb | 0.06 | 0.02 | 2.58 | <1.00 x 10^-7^ | 0.11 | 0.04 | 3.13 | <1.00 x 10^-7^ |
| >8Mb | 0.05 | 0.02 | 3.12 | <1.00 x 10^-7^ | 0.09 | 0.03 | 3.43 | <1.00 x 10^-7^ |
| >9Mb | 0.04 | 0.01 | 3.17 | <1.00 x 10^-7^ | 0.08 | 0.02 | 3.48 | <1.00 x 10^-7^ |
| >10Mb | 0.03 | 0.01 | 2.74 | <1.00 x 10^-7^ | 0.06 | 0.02 | 3.14 | 3.60 x 10^-6^ |
