# Supplementary material for: Cooperative Genome-Wide Analysis Shows Increased Homozygosity in Early Onset Parkinson's Disease
Source: PLoS One. 2012 Mar 12;7(3):e28787. doi: 10.1371/journal.pone.0028787 (PMC3299635; doi:10.1371/journal.pone.0028787)
Supplement: Table S4 — Logistic models. a) Logistic models with proportion of samples with at least one ROH of a given minimum size as independent variable, and phenotype as dependent variable. b) Logistic models with rate of ROH of a given minimum size as independent variable, and phenotype as dependent variable. (Covariates - Model 1: unadjusted; Model 2: f; Model 3: f, age; Model 4: f, age, MDS) (DOC) [file pone.0028787.s010.doc]

| a) | **Model 1** | | **Model 2** | | **Model 3** | | **Model 4** | |
| --- | --- | --- | --- | --- | --- | --- | --- | --- |
| **Size** | *P value* | *OR* | *P value* | *OR* | *P value* | *OR* | *P value* | *OR* |
|  |  | *(95%CI)* |  | *(95% CI)* |  | *(95% CI)* |  | *(95% CI)* |
| >1Mb | NA | NA | NA | NA | NA | NA | NA | NA |
| >2Mb | n.s. | 0.85 | n.s. | 0.88 | n.s. | 0.88 | n.s. | 0.88 |
|  |  | (0.71-1.01) |  |  |  | (0.71-1.12) |  | (0.70-1.12) |
| >3Mb | 0.03 | 1.14 | 0.01 | 1.17 | 0.03 | 1.18 | n.s. | 1.15 |
|  |  | (1.02-1.28) |  | (0.74-1.06) |  | (1.02-1.37) |  | (0.99-1.33) |
| >4Mb | 4.40 x 10^-3^ | 1.24 | 5.64 x 10^-4^ | 1.3 | 1.47 x 10^-3^ | 1.34 | 0.01 | 1.3 |
|  |  | (1.02-1.28) |  | (1.05-1.32) |  | (1.12-1.61) |  | (1.08-1.56) |
| >5Mb | 1.61 x 10^-7^ | 1.65 | 1.43 x 10^-9^ | 1.8 | 6.91 x 10^-6^ | 1.7 | 4.44 x 10^-5^ | 1.63 |
|  |  | (1.07-1.43) |  | (1.12-1.50) |  | (1.34-2.13) |  | (1.28-2.05) |
| >6Mb | 2.31 x 10^-9^ | 2.03 | 1.98 x 10^-12^ | 2.34 | 5.14 x 10^-7^ | 2.06 | 1.19 x 10^-5^ | 1.91 |
|  |  | (1.37-1.99) |  | (1.48-2.17) |  | (1.55-2.72) |  | (1.42-2.53) |
| >7Mb | 7.25 x 10^-13^ | 2.68 | <2.00 x 10^-16^ | 3.25 | 1.25 x 10^-9^ | 2.74 | 3.25 x 10^-7^ | 2.39 |
|  |  | (2.04-3.50) |  | (1.84-2.95) |  | (1.96-3.76) |  | (1.70-3.31) |
| >8Mb | 3.95 x 10^-14^ | 3.23 | <2.00 x 10^-16^ | 4.02 | 2.68 x 10^-9^ | 3.06 | 7.73 x 10^-7^ | 2.59 |
|  |  | (2.38-4.37) |  | (2.46-4.27) |  | (2.1-4.39) |  | (1.76-3.74) |
| >9Mb | 6.29 x 10^-13^ | 3.27 | <2.00 x 10^-16^ | 4.12 | 7.91 x 10^-9^ | 3.15 | 2.35 x 10^-6^ | 2.62 |
|  |  | (2.36-4.51) |  | (2.94-5.73) |  | (2.12-4.62) |  | (1.74-3.88) |
| >10Mb | 2.67 x 10^-8^ | 2.8 | 8.74 x 10^-12^ | 3.67 | 2.76 x 10^-5^ | 2.56 | 1.35 x 10^-3^ | 2.09 |
|  |  | (1.93-4.00) |  | (2.51-5.30) |  | (1.63-3.94) |  | (1.32-3.25) |

| b) | **Model 1** | | **Model 2** | | **Model 3** | | **Model 4** | |
| --- | --- | --- | --- | --- | --- | --- | --- | --- |
| **Size** | *P value* | *OR(95% CI)* | *P value* | *OR* | *P value* | *OR* | *P value* | *OR* |
|  |  |  |  | *(95% CI)* |  | *(95% CI)* |  | *(95% CI)* |
| >1Mb | n.s. | 0.98 | n.s. | 0.99 | n.s. | 1 | n.s. | 1 |
|  |  | (0.97-0.99) |  | (0.97-1.00) |  | (0.98-1.01) |  | (0.98-1.01) |
| >2Mb | n.s. | 0.99 | n.s. | 1.01 | n.s. | 1.01 | n.s. | 1 |
|  |  | (0.96-1.03) |  | (0.98-1.05) |  | (0.97-1.06) |  | (0.96-1.04) |
| >3Mb | 1.97 x 10^-6^ | 1.14 | 1.18 x 10^-10^ | 1.21 | 1.25 x 10^-4^ | 1.14 | 0.01 | 1.10 |
|  |  | (1.08-1.21) |  | (1.14-1.28) |  | (1.07-1.22) |  | (1.03-1.18) |
| >4Mb | 6.10 x 10^-7^ | 1.21 | 4.40 x 10^-12^ | 1.31 | 4.13 x 10^-4^ | 1.18 | 0.01 | 1.12 |
|  |  | (1.12-1.30) |  | (1.21-1.41) |  | (1.08-1.29) |  | (1.02-1.23) |
| >5Mb | 2.44 x 10^-8^ | 1.3 | 2.20 x 10^-14^ | 1.44 | 3.15 x 10^-4^ | 1.22 | 0.01 | 1.16 |
|  |  | (1.19-1.43) |  | (1.31-1.58) |  | (1.10-1.37) |  | (1.04-1.30) |
| >6Mb | 1.20 x 10^-8^ | 1.38 | 7.99 x 10^-15^ | 1.57 | 9.70 x 10^-5^ | 1.30 | 4.72 x 10^-3^ | 1.21 |
|  |  | (1.24-1.55) |  | (1.40-1.76) |  | (1.14-1.48) |  | (1.06-1.38) |
| >7Mb | 1.99 x 10^-8^ | 1.44 | 2.89 x 10^-14^ | 1.65 | 1.49 x 10^-4^ | 1.32 | 0.01 | 1.22 |
|  |  | (1.27-1.64) |  | (1.45-1.89) |  | (1.15-1.54) |  | (1.06-1.42) |
| >8Mb | 7.14 x 10^-8^ | 1.5 | 4.89 x 10^-13^ | 1.74 | 4.17 x 10^-4^ | 1.35 | 0.01 | 1.23 |
|  |  | (1.30-1.75) |  | (1.50-2.03) |  | (1.14-1.59) |  | (1.05-1.46) |
| >9Mb | 1.38 x 10^-7^ | 1.55 | 1.03 x 10^-12^ | 1.83 | 4.93 x 10^-4^ | 1.39 | 0.01 | 1.27 |
|  |  | (1.32-1.83) |  | (1.55-2.17) |  | (1.16-1.68) |  | (1.06-1.54) |
| >10Mb | 1.68 x 10^-5^ | 1.49 | 8.12 x 10^-10^ | 1.8 | 7.16 x 10^-3^ | 1.33 | n.s. | 1.21 |
|  |  | (1.25-1.80) |  | (1.50-2.18) |  | (1.09-1.64) |  | (0.98-1.49) |
