# Supplementary material for: Cooperative Genome-Wide Analysis Shows Increased Homozygosity in Early Onset Parkinson's Disease
Source: PLoS One. 2012 Mar 12;7(3):e28787. doi: 10.1371/journal.pone.0028787 (PMC3299635; doi:10.1371/journal.pone.0028787)
Supplement: Table S5 — Logistic models. a) Logistic models with proportion of samples with at least one rare ROH of a given minimum size as independent variable, and phenotype as dependent variable. b) Logistic models with rate of rare ROH of a given minimum size as independent variable, and phenotype as dependent variable. (Covariates - Model 1: unadjusted; Model 2: f; Model 3: f, age; Model 4: f, age, MDS) (DOC) [file pone.0028787.s011.doc]

| a) | **Model 1** | | **Model 2** | | **Model 3** | |
| --- | --- | --- | --- | --- | --- | --- |
| **Size** | *P value* | *Odds Ratio*  *(95% CI)* | *P value* | *Odds Ratio*  *(95% CI)* | *P value* | *Odds Ratio*  *(95% CI)* |
| >2Mb | n.s. | 0.91 (0.82-1.02) | n.s. | 0.95 (0.85-1.07) | n.s. | 1.01 (0.88-1.17) |
| >3Mb | n.s. | 1.10 (0.96-1.26) | 0.03 | 1.16 (1.01-1.34) | 4.25 x 10^-3^ | 1.28 (1.08-1.52) |
| >4Mb | 5.35 x 10^-6^ | 1.51 (1.26-1.81) | 3.79 x 10^-8^ | 1.66 (1.38-1.99) | 2.32 x 10^-6^ | 1.70 (1.36-2.10) |
| >5Mb | 1.76 x 10^-8^ | 1.79 (1.46-2.18) | 6.05 x 10^-11^ | 1.98 (1.61-2.42) | 5.96 x 10^-7^ | 1.87 (1.46-2.28) |
| >6Mb | 2.31 x 10^-9^ | 2.03 (1.37-1.99) | 1.98 x 10^-12^ | 2.34 (1.48-2.17) | 5.14 x 10^-7^ | 2.06 (1.55-2.72) |
| >7Mb | 7.25 x 10^-13^ | 2.68 (2.04-3.50) | <2.00 x 10^-16^ | 3.25 (1.84-2.95) | 1.25 x 10^-9^ | 2.74 (1.96-3.76) |
| >8Mb | 3.95 x 10^-14^ | 3.23 (2.38-4.37) | <2.00 x 10^-16^ | 4.02 (2.46-4.27) | 2.68 x 10^-9^ | 3.06 (2.10-4.39) |
| >9Mb | 6.29 x 10^-13^ | 3.27 (2.36-4.51) | <2.00 x 10^-16^ | 4.12 (2.94-5.73) | 7.91 x 10^-9^ | 3.15 (2.12-4.62) |
| >10Mb | 2.67 x 10^-8^ | 2.80 (1.93-4.00) | 8.74 x 10^-12^ | 3.67 (2.51-5.30) | 2.76 x 10^-5^ | 2.56 (1.63-3.94) |

| b) | **Model 1** | | **Model 2** | | **Model 3** | |
| --- | --- | --- | --- | --- | --- | --- |
| **Size** | *P value* | *Odds Ratio*  *(95% CI)* | *P value* | *Odds Ratio*  *(95% CI)* | *P value* | *Odds Ratio*  *(95% CI)* |
| >2Mb | 0.01 | 1.06 (1.01-1.11) | 5.27 x 10^-5^ | 1.11 (1.05-1.16) | 0.01 | 1.07 (1.01-1.13) |
| >3Mb | 1.93 x 10^-6^ | 1.17 (1.09-1.24) | 1.23 x 10^-11^ | 1.25 (1.17-1.34) | 3.33 x 10^-5^ | 1.18 (1.09-1.27) |
| >4Mb | 7.55 x 10^-8^ | 1.25 (1.15-1.36) | 5.66 x 10^-14^ | 1.37 (1.27-1.50) | 1.92 x 10^-4^ | 1.20 (1.09-1.33) |
| >5Mb | 2.07 x 10^-8^ | 1.31 (1.19-1.45) | 1.32 x 10^-14^ | 1.46 (1.32-1.61) | 2.65 x 10^-4^ | 1.23 (1.10-1.38) |
| >6Mb | 1.20 x 10^-8^ | 1.38 (1.24-1.55) | 7.99 x 10^-15^ | 1.57 (1.40-1.76) | 9.70 x 10^-5^ | 1.30 (1.14-1.48) |
| >7Mb | 1.99 x 10^-8^ | 1.44 (1.27-1.64) | 2.89 x 10^-14^ | 1.65 (1.45-1.89) | 1.49 x 10^-4^ | 1.32 (1.15-1.54) |
| >8Mb | 7.14 x 10^-8^ | 1.50 (1.30-1.75) | 4.89 x 10^-13^ | 1.74 (1.50-2.03) | 4.17 x 10^-4^ | 1.35 (1.14-1.59) |
| >9Mb | 1.38 x 10^-7^ | 1.55 (1.32-1.83) | 1.03 x 10^-12^ | 1.83 (1.55-2.17) | 4.93 x 10^-4^ | 1.39 (1.16-1.68) |
| >10Mb | 1.68 x 10^-5^ | 1.49 (1.25-1.80) | 8.12 x 10^-10^ | 1.80 (1.50-2.18) | 7.16 x 10^-3^ | 1.33 (1.09-1.64) |
