# Supplementary material for: Cooperative Genome-Wide Analysis Shows Increased Homozygosity in Early Onset Parkinson's Disease
Source: PLoS One. 2012 Mar 12;7(3):e28787. doi: 10.1371/journal.pone.0028787 (PMC3299635; doi:10.1371/journal.pone.0028787)
Supplement: Table S6 — Burden analysis following the exclusion of samples with ROH>8 Mb in size. a) Proportion of samples with ROH of a given minimum size. b) Rate of ROH of a given minimum size. (DOC) [file pone.0028787.s012.doc]

|  | a)       Proportion | | | | b)       Rate | | | |
| --- | --- | --- | --- | --- | --- | --- | --- | --- |
| **Size** | **EOPD** | **Controls** | **Ratio** | **P value** | **EOPD** | **Controls** | **Ratio** | **P value** |
| >2Mb | 0.88 | 0.90 | 0.98 | 0.99 | 2.07 | 2.21 | 0.94 | 1.00 |
| >3Mb | 0.40 | 0.38 | 1.03 | 0.22 | 0.49 | 0.48 | 1.02 | 0.30 |
| >4Mb | 0.15 | 0.14 | 1.01 | 0.45 | 0.16 | 0.16 | 1.04 | 0.31 |
| >5Mb | 0.07 | 0.06 | 1.18 | 0.09 | 0.07 | 0.06 | 1.20 | 0.06 |
| >6Mb | 0.03 | 0.02 | 1.16 | 0.23 | 0.03 | 0.02 | 1.24 | 0.13 |
| >7Mb | 0.01 | 0.01 | 1.43 | 0.15 | 0.01 | 0.01 | 1.43 | 0.15 |
