# Supplementary material for: Cooperative Genome-Wide Analysis Shows Increased Homozygosity in Early Onset Parkinson's Disease
Source: PLoS One. 2012 Mar 12;7(3):e28787. doi: 10.1371/journal.pone.0028787 (PMC3299635; doi:10.1371/journal.pone.0028787)
Supplement: Table S7 — Burden analysis following the exclusion of ROH>8 Mb in size. a) Proportion of samples with ROH of a given minimum size. b) Rate of ROH of a given minimum size. (DOC) [file pone.0028787.s013.doc]

|  | a)       Proportion | | | | b)       Rate | | | |
| --- | --- | --- | --- | --- | --- | --- | --- | --- |
| **Size** | **EOPD** | **Controls** | **Ratio** | **P value** | **EOPD** | **Controls** | **Ratio** | **P value** |
| >2Mb | 0.88 | 0.90 | 0.98 | 0.98 | 2.15 | 2.23 | 0.96 | 0.98 |
| >3Mb | 0.42 | 0.39 | 1.08 | 0.02 | 0.56 | 0.49 | 1.13 | 2.90 x 10^-3^ |
| >4Mb | 0.16 | 0.15 | 1.09 | 0.10 | 0.20 | 0.17 | 1.21 | 0.01 |
| >5Mb | 0.08 | 0.06 | 1.29 | 0.01 | 0.10 | 0.07 | 1.46 | 5.00 x 10^-4^ |
| >6Mb | 0.04 | 0.03 | 1.45 | 0.01 | 0.05 | 0.03 | 1.74 | 6.00 x 10^-4^ |
| >7Mb | 0.02 | 0.01 | 1.90 | 0.01 | 0.02 | 0.01 | 2.23 | 1.60 x 10^-3^ |
