# Supplementary material for: Cooperative Genome-Wide Analysis Shows Increased Homozygosity in Early Onset Parkinson's Disease
Source: PLoS One. 2012 Mar 12;7(3):e28787. doi: 10.1371/journal.pone.0028787 (PMC3299635; doi:10.1371/journal.pone.0028787)
Supplement: Table S9 — Burden analysis following the exclusion of samples with rare ROH>2 Mb size across known PARK loci. a) Proportion of samples with rare ROH of a given minimum size. b) Rate of rare ROH of a given minimum size. (DOC) [file pone.0028787.s015.doc]

|  | 1. Proportion | | | | 1. Rate | | | |
| --- | --- | --- | --- | --- | --- | --- | --- | --- |
| **Size** | **EOPD** | **Controls** | **Ratio** | **P value** | **EOPD** | **Controls** | **Ratio** | **P value** |
| >2Mb | 0.51 | 0.53 | 0.96 | 0.94 | 0.86 | 0.81 | 1.07 | 0.03 |
| >3Mb | 0.21 | 0.20 | 1.05 | 0.22 | 0.33 | 0.24 | 1.36 | 1.90 x 10^-5^ |
| >4Mb | 0.11 | 0.08 | 1.41 | 6.40 x 10^-5^ | 0.19 | 0.10 | 1.84 | <1.00 x 10^-6^ |
| >5Mb | 0.09 | 0.05 | 1.62 | 3.00 x 10^-6^ | 0.15 | 0.07 | 2.08 | <1.00 x 10^-6^ |
| >6Mb | 0.07 | 0.04 | 1.81 | 3.00 x 10^-6^ | 0.11 | 0.05 | 2.41 | <1.00 x 10^-6^ |
| >7Mb | 0.05 | 0.02 | 2.37 | <1.00 x 10^-6^ | 0.09 | 0.03 | 2.90 | <1.00 x 10^-6^ |
| >8Mb | 0.04 | 0.01 | 2.96 | <1.00 x 10^-6^ | 0.07 | 0.02 | 3.27 | <1.00 x 10^-6^ |
| >9Mb | 0.04 | 0.01 | 3.05 | <1.00 x 10^-6^ | 0.06 | 0.02 | 3.27 | 2.00 x 10^-6^ |
| >10Mb | 0.03 | 0.01 | 2.43 | 1.30 x 10^-5^ | 0.05 | 0.02 | 2.83 | 6.10 x 10^-5^ |
