# Supplementary material for: Cooperative Genome-Wide Analysis Shows Increased Homozygosity in Early Onset Parkinson's Disease
Source: PLoS One. 2012 Mar 12;7(3):e28787. doi: 10.1371/journal.pone.0028787 (PMC3299635; doi:10.1371/journal.pone.0028787)
Supplement: Table S10 — Logistic models following the exclusion of samples with all ROH>2 Mb across known PARK loci. a) Logistic models with proportion of samples with at least one ROH of a given minimum size as independent variable, and phenotype as dependent variable. b) Logistic models with rate of all ROH of a given minimum size as independent variable, and phenotype as dependent variable. (Covariates - Model 1: unadjusted; Model 2: f; Model 3: f, age; Model 4: f, age, MDS) (DOC) [file pone.0028787.s016.doc]

| a) | **Model 1** | | **Model 2** | | **Model 3** | | **Model 4** | |
| --- | --- | --- | --- | --- | --- | --- | --- | --- |
| **Size** | *P value* | *Odds Ratio*  *(95%CI)* | *P value* | *Odds Ratio*  *(95%CI)* | *P value* | *Odds Ratio*  *(95%CI)* | *P value* | *Odds Ratio*  *(95%CI)* |
| >2Mb | 0.05 | 0.84  (0.70-1.01) | n.s. | 0.88  (0.73-1.05) | n.s. | 0.87  (0.69-1.10) | n.s. | 0.87  (0.69-1.10) |
| >3Mb | 0.05 | 1.12  (1.00-1.26) | 0.02 | 1.16  (1.03-1.30) | 0.05 | 1.16  (1.00-1.35) | n.s | 1.13  (0.97-1.31) |
| >4Mb | 0.02 | 1.20  (1.03-1.40) | 4.00 x 10^-3^ | 1.25  (1.07-1.46) | 4.49 x 10^-3^ | 1.31  (1.09-1.58) | 0.01 | 1.28  (1.06-1.55) |
| >5Mb | 7.87 x 10^-6^ | 1.57  (1.28-1.90) | 2.11 x 10^-7^ | 1.69  (1.38-2.06) | 4.30 x 10^-5^ | 1.65  (1.29-2.09) | 1.30 x 10^-4^ | 1.61  (1.26-2.04) |
| >6Mb | 8.50 x 10^-7^ | 1.86  (1.45-2.38) | 4.36 x 10^-9^ | 2.12  (1.65-2.72) | 1.52 x 10^-5^ | 1.94  (1.43-2.62) | 1.19 x 10^-4^ | 1.83  (1.33-2.47) |
| >7Mb | 2.11 x 10^-9^ | 2.44  (1.81-3.25) | 2.43 x 10^-12^ | 2.89  (2.14-3.88) | 7.91 x 10^-8^ | 2.60  (1.82-3.67) | 4.10 x 10^-6^ | 2.32  (1.61-3.29) |
| >8Mb | 3.15 x 10^-11^ | 3.05  (2.18-4.22) | 2.51 x 10^-14^ | 3.70  (2.63-5.16) | 2.23 x 10^-8^ | 3.07  (2.06-4.53) | 1.77 x 10^-6^ | 2.66  (1.76-3.95) |
| >9Mb | 2.04 x 10^-10^ | 3.13  (2.19-4.43) | 2.34 x 10^-13^ | 3.84  (2.67-5.49) | 6.72 x 10^-8^ | 3.19  (2.07-4.82) | 5.43 x 10^-6^ | 2.72  (1.75-4.14) |
| >10Mb | 8.71 x 10^-6^ | 2.47  (1.64-3.66) | 2.54 x 10^-8^ | 3.19  (2.10-4.77) | 3.33 x 10^-4^ | 2.41  (1.47-3.85) | 0.01 | 2.01  (1.21-3.24) |

| b) | **Model 1** | | **Model 2** | | **Model 3** | | **Model 4** | |
| --- | --- | --- | --- | --- | --- | --- | --- | --- |
| **Size** | *P value* | *Odds Ratio*  *(95%CI)* | *P value* | *Odds Ratio*  *(95%CI)* | *P value* | *Odds Ratio*  *(95%CI)* | *P value* | *Odds Ratio*  *(95%CI)* |
| >2Mb | n.s. | 0.99  (0.97-0.99) | n.s. | 1.01  (0.97-1.05) | n.s. | 1.01  (0.96-1.06) | n.s. | 1.00  (0.95-1.05) |
| >3Mb | 1.97 x 10^-5^ | 1.14  (0.95-1.02) | 1.19 x 10^-8^ | 1.21  (1.13-1.29) | 1.09 x 10^-4^ | 1.16  (1.08-1.25) | 2.90 x 10^-3^ | 1.12  (1.04-1.21) |
| >4Mb | 5.79 x 10^-6^ | 1.21  (1.08-1.22) | 6.46 x 10^-10^ | 1.31  (1.20-1.43) | 2.42 x 10^-4^ | 1.21  (1.09-1.34) | 0.01 | 1.16  (1.04-1.29) |
| >5Mb | 3.32 x 10^-7^ | 1.32  (1.12-1.32) | 6.26 x 10^-12^ | 1.46  (1.31-1.63) | 1.83 x 10^-4^ | 1.28  (1.12-1.45) | 2.69 x 10^-3^ | 1.22  (1.07-1.39) |
| >6Mb | 1.97 x 10^-7^ | 1.42  (1.19-1.48) | 1.84 x 10^-12^ | 1.61  (1.41-1.84) | 5.70 x 10^-5^ | 1.37  (1.18-1.60) | 1.66 x 10^-3^ | 1.28  (1.10-1.49) |
| >7Mb | 4.25 x 10^-7^ | 1.47  (1.25-1.62) | 7.55 x 10^-12^ | 1.70  (1.46-1.98) | 9.30 x 10^-5^ | 1.41  (1.19-1.68) | 2.82 x 10^-3^ | 1.30  (1.09-1.55) |
| >8Mb | 4.52 x 10^-7^ | 1.57  (1.32-1.87) | 2.05 x 10^-11^ | 1.82  (1.53-2.18) | 1.18 x 10^-4^ | 1.47  (1.21-1.79) | 3.50 x 10^-3^ | 1.34  (1.10-1.63) |
| >9Mb | 9.43 x 10^-7^ | 1.61  (1.34-1.96) | 4.16 x 10^-11^ | 1.90  (1.58-2.32) | 1.79 x 10^-4^ | 1.52  (1.22-1.90) | 3.75 x 10^-3^ | 1.38  (1.11-1.72) |
| >10Mb | 7.80 x 10^-5^ | 1.52  (1.24-1.88) | 1.45 x 10^-8^ | 1.84  (1.49-2.29) | 0.01 | 1.41  (1.11-1.80) | 0.04 | 1.28  (1.00-1.64) |
