# Supplementary material for: Cooperative Genome-Wide Analysis Shows Increased Homozygosity in Early Onset Parkinson's Disease
Source: PLoS One. 2012 Mar 12;7(3):e28787. doi: 10.1371/journal.pone.0028787 (PMC3299635; doi:10.1371/journal.pone.0028787)
Supplement: Table S12 — Gene-based homozygosity mapping results of the most prominent PARK genes. P = uncorrected p value. P* = p value corrected for multiple testing using 100,000 case/control status permutations (DOC) [file pone.0028787.s018.doc]

| **Chromosome** | **Gene** | **P** | **P*** |
| --- | --- | --- | --- |
| 1 | *PARK7* | 1.00 | 1.00 |
| 1 | *ATP13A2* | 0.61 | 1.00 |
| 1 | *PINK1* | 1.00 | 1.00 |
| 4 | *SNCA* | 0.41 | 0.98 |
| 6 | *PARK2* | 0.01 | 0.04 |
| 12 | *LRRK2* | 0.17 | 0.51 |
| 15 | *SPG11* | 0.14 | 0.67 |
| 16 | *FA2H* | 0.21 | 0.85 |
| 21 | *PDXK* | 0.53 | 1.00 |
| 22 | *FBXO7* | 0.03 | 0.26 |
| 22 | *PLA2G6* | 0.14 | 0.67 |

P = uncorrected p value

P* = p value corrected for multiple testing using 100,000 case/control status permutations
